# Standardizing Postoperative Rehabilitation Protocols for the Tri-Service: A Consensus Meeting Hosted by the Musculoskeletal Injury Rehabilitation Research for Operational Readiness Organization

**DOI:** 10.1093/milmed/usaa207

**Published:** 2020-12-30

**Authors:** Brad Isaacson, Mae Miranda, Nelson Hager, Linzie Wagner, Sydney West, Whitley Lucio, John Heller, Robert Dalgarno, Jonathan F Dickens, Eric Schoomaker, Paul Pasquina

**Affiliations:** Department of Physical Medicine & Rehabilitation, Musculoskeletal Injury Rehabilitation Research for Operational Readiness (MIRROR), Uniformed Services University, Bethesda, MD 20814; The Geneva Foundation, Tacoma, WA 98402; Walter Reed National Military Medical Center, Bethesda, MD 20814; Department of Physical Medicine & Rehabilitation, Musculoskeletal Injury Rehabilitation Research for Operational Readiness (MIRROR), Uniformed Services University, Bethesda, MD 20814; Walter Reed National Military Medical Center, Bethesda, MD 20814; Department of Physical Medicine & Rehabilitation, Musculoskeletal Injury Rehabilitation Research for Operational Readiness (MIRROR), Uniformed Services University, Bethesda, MD 20814; The Geneva Foundation, Tacoma, WA 98402; Department of Physical Medicine & Rehabilitation, Musculoskeletal Injury Rehabilitation Research for Operational Readiness (MIRROR), Uniformed Services University, Bethesda, MD 20814; The Geneva Foundation, Tacoma, WA 98402; Department of Physical Medicine & Rehabilitation, Musculoskeletal Injury Rehabilitation Research for Operational Readiness (MIRROR), Uniformed Services University, Bethesda, MD 20814; The Geneva Foundation, Tacoma, WA 98402; Department of Physical Medicine & Rehabilitation, Musculoskeletal Injury Rehabilitation Research for Operational Readiness (MIRROR), Uniformed Services University, Bethesda, MD 20814; The Geneva Foundation, Tacoma, WA 98402; Department of Physical Medicine & Rehabilitation, Musculoskeletal Injury Rehabilitation Research for Operational Readiness (MIRROR), Uniformed Services University, Bethesda, MD 20814; The Geneva Foundation, Tacoma, WA 98402; Department of Physical Medicine & Rehabilitation, Musculoskeletal Injury Rehabilitation Research for Operational Readiness (MIRROR), Uniformed Services University, Bethesda, MD 20814; Walter Reed National Military Medical Center, Bethesda, MD 20814; Department of Physical Medicine & Rehabilitation, Musculoskeletal Injury Rehabilitation Research for Operational Readiness (MIRROR), Uniformed Services University, Bethesda, MD 20814; Department of Military and Emergency Medicine, Uniformed Services University, Bethesda, MD; Department of Physical Medicine & Rehabilitation, Musculoskeletal Injury Rehabilitation Research for Operational Readiness (MIRROR), Uniformed Services University, Bethesda, MD 20814; Walter Reed National Military Medical Center, Bethesda, MD 20814

## OVERVIEW

The cost of health care in the United States has increased exponentially over the past 60 years, soaring from $27.2 billion in 1960 (5% of gross domestic product) and $147 per resident to $3.5 trillion (17.9% of gross domestic product) and $11 000 per resident in 2017.^1^ Health care expenditures continue to rise significantly faster than the median household income, and this poses a financial strain for patients, providers, and the health care system alike. Removing unnecessary variation through evidence-based medicine is critical to improving outcomes and making care more affordable. This may be accomplished through standardized protocols, order sets, and check lists, with positive results previously demonstrated for obstetrics/gynecology,^2^ critical care,^3^ pediatrics,^4^ gastrointestinal surgery,^5^ orthopedics,^6^ and rehabilitation.^7^ Effective communication with a multidisciplinary team has also shown to enhance quality of treatment, reduce complications, and decrease postoperative issues.^8^^,^^9^ Although treatment normalization is a pragmatic solution for removing health care waste, evidence supports that even when guidelines are available, only two-thirds of patients receive the recommended care, and another quarter get treatment that may be unnecessary and/or harmful.^10^

One of the greatest challenges for improving patient safety is determining how to implement evidence-based care and deploy it uniformly across hospitals and clinics.^11^ This problem is often exacerbated by geographic separation between clinical locations, complexity of information technology/electronic medical record data systems, cultural barriers, habitual practicing approaches to medicine, disagreement on the preferred treatments, limited time with patients to discuss comorbidities, changing shifts of health care workers, etc. The Military Health System (MHS) faces these challenges, and must also provide aid for Service Members in over 150 countries, transition care between the Department of Defense (DoD), Department of Veterans Affairs, and public sector, and offer treatment after incidents of polytrauma sustained in combat. Streamlining care within the military is difficult, as a single patient may experience hundreds of caregivers during their tenure.

In 2016, the National Defense Authorization Act formalized the Joint Trauma System (JTS) within the DoD to align all military treatment facilities (MTFs) under a unified standard rather than having separate protocols for the Tri-Service (Army, Navy, and Air Force). The mission of the JTS is “to improve trauma readiness and outcomes through evidence-driven performance improvement. The JTS vision is that every Soldier, Sailor, Airman and Marine injured on the battlefield or in any theater of operations will be provided with the optimum chance for survival and maximum potential for functional recovery.”^12^ This same level of medical/operational rigor is needed for all clinical domains, especially as the conflicts in Iraq and Afghanistan drawdown. Preventing nonbattle injuries is key to enhancing readiness, resilience, and removing unnecessary health care waste. The most obvious next steps are augmenting the prevention, diagnosis, and treatment of musculoskeletal injuries (MSIs) within the military and civilian sectors.

## THE FORMATION OF A NEW MUSCULOSKELETAL ORGANIZATION

MSIs affect approximately 800 000 Service Members annually and result in 25 million days of limited duty.^13^ These conditions are the primary reasons for medical discharge and downgrade and result in 34% of medical evacuations from theater.^14^ The direct and indirect costs associated with MSIs challenge an already strained medical system, cost taxpayers billions of dollars, and have the potential to threaten our national security. Given the strict requirements for physical fitness in the military, and effect of MSI on combat readiness, the Defense Health Agency (DHA) supported the creation of the Musculoskeletal Injury Rehabilitation Research for Operational Readiness (MIRROR) organization in 2019 to provide critical infrastructure, operational, and research support to advance the treatment and preventive care for Service Members with noncombat-related MSI. Headquartered at the Uniformed Services University of the Health Sciences in the Department of Physical Medicine & Rehabilitation, MIRROR coordinates interservice partnerships with the primary MTFs, as well as other sites that experience a high volume of MSI, but lack a robust infrastructure to conduct rigorous clinical and translational studies (www.mirrorusuhs.org). MIRROR focuses on 4 research areas, which include the following: (1) identification and treatment of MSI risk factors and comorbid disorders, (2) optimization of standard of care practices for the treatment and rehabilitation of MSI, (3) establishment of strategies to mitigate injury occurrence, and (4) application of new technologies in preventative and rehabilitative care. MIRROR disseminates high-value knowledge products, and in January 2020, the organization held its first annual “Post-Operative Rehabilitation Protocol Consensus Meeting for the Tri-Service” to streamline guidelines for rehabilitative care. Much like the JTS, this symposium was directed at reaching a unified strategy across the military. Uniquely, our team focused on MSIs for both preparedness and recovery. The deliberations of the event are detailed in the following section as a case example for other similar workgroups to deliver value products to the health care system.

## THE POSTOPERATIVE REHABILITATION PROTOCOLS FOR THE TRI-SERVICE EVENT

More than 50 military leaders, clinicians, scientists, and subject-matter experts within the physical therapy, orthopedics, physical medicine and rehabilitation, and pain management fields converged for an all-day consensus meeting to standardize postoperative care for Service Members. The first-of-its-kind assembly sought to identify and normalize best practices within the Army, Navy, and Air Force (Fig. I, table for Fig. I). Eleven protocols were selected based on the frequency of the procedures performed; ones that would lend themselves to standardization, and actionable ones that could deliver immediate value to the MHS, DHA, and general public (Table I). The consensus meeting also highlighted areas for future protocol collaboration, as well as gaps in optimizing postoperative physical therapy guidelines to design, fund, and/or execute future relevant research studies in this field. The meeting started with the full attendees together in a singular room, followed by 4 separate breakout sessions held for the specific anatomical areas being evaluated (eg, hip, knee, ankle, and shoulder). Each session was proctored by an orthopedic surgeon and physical therapist who guided the discussion as a dyad. A transcription service was used to capture the dialogue among participants and to understand the dissimilarities in practice across the Tri-Service. Four key themes emerged at the conclusion of the meeting and they are organized as follows.

**FIGURE 1:**
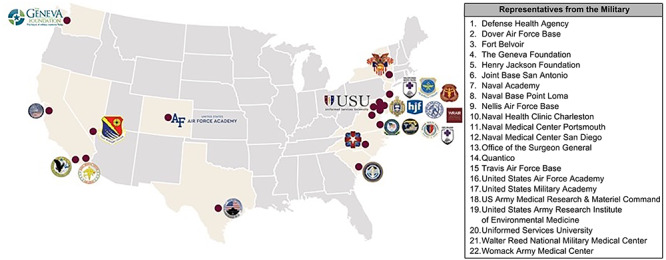
Sites Represented at the MIRROR Sponsored Postoperative Rehabilitation Consensus Meeting.

**TABLE I:** Protocols Standardized

Shoulder	
1.	Accelerated postoperative shoulder rehabilitation (subacromial decompression, distal clavicle resection, debridement)
2.	Bicep tenodesis
3.	Rotator cuff repair
4.	Anterior/posterior capsulorrhaphy and/or arthroscopic reverse Bankart repair
Knee	
5.	Postoperative patellar tendon repair
6.	Arthroscopic knee surgery
7.	Anterior cruciate ligament reconstruction
8.	Meniscus repair
Ankle	
9.	Ankle reconstruction/modified Brostrom
10.	Achilles tendon repair
Hip	
11.	Arthroscopic hip rehabilitation guidelines with labral repair and arthroscopy

### Theme Number 1: Addressing the Importance of Standardization

Participants from the Tri-Service meeting universally agreed that there has been a need to standardize medical care in order to improve patient-centered outcomes. The military in particular is a unique case, as the DoD realignment has resulted in consolidated bases, making interactions between providers and patients from the different branches more common. Barriers that currently exist include the use of different vernaculars/acronyms, distinct and different cultural norms, being unaware of all of the important clinical/operational stakeholders, etc. In order to truly standardize care, protocols must be written and verbalized in a manner that all parties can understand and act upon. Documents should also be stored in a common repository, whether it be through a World Wide Web page or shared drive, so that they are easily accessible for training, educating other front-line providers, and sharing with senior leadership. Last, but not least, there are hundreds of different outcome measures referenced in the peer-reviewed literature, and selecting several high-value ones in the future is essential. This facilitates the creation of longitudinal data sets, research translation from benchtop to bedside, and leverages platforms like the Military Orthopaedics Tracking Injuries and Outcomes Network (MOTION) for monitoring performance, readiness, and resilience.

### Theme Number 2: How Do We Characterize Pain?

Assessing pain is traditionally recorded on a 0 to 10 scale, and patients are prescribed medications based on their selection and tolerance. However, focusing on just the intensity of pain is misleading, and the biopsychosocial phenomenon must be considered. More specifically, someone may say their pain is a “7 out of 10,” but that may not align with their recent improvements with their home life, sleep, mood, appetite, etc. Pain discussions between a patient and their provider need to be framed around “how” the pain is impacting their ability to do what they need to, as well as better ways to develop a specific treatment plan for achieving the functional outcomes desired. This is especially relevant following MSI, since pain management immediately after an injury has been associated with impacts on long-term recovery, rehabilitation, etc. In order to successfully return a Service Member to duty, or transition him/her to the civilian community, effective and coordinated pain management plans must be developed early within the process. The focus must be on functional restoration and achievement of key milestones and not on pharmacologic (in particular, opioid) treatments for pain management in the acute and subacute phases of recovery.

### Theme Number 3: Creating a Framework for a New Protocol

When standardizing protocols, the methodology should include the following: (1) a common set of goals that the patient wishes to achieve based on their injury and rehabilitation regimen designed with clinical oversight, (2) agreed upon restrictions to ensure proper recovery, (3) established milestones and deliverables (eg, the period to mobilization, partial weight-bearing, etc.) for measuring progress and motivating a patient, and (4) setting follow-up criteria to prevent re-injury. It was also recognized that MTFs where there were strong and collaborative professional relationships between all health care workers (eg, surgeons, physical therapists, occupational therapists, nurses, etc.), compliance and adherence would be the highest. However, there was a common understanding that each injury is unique and treatment should include other comorbidities. Thus, obtaining a clear baseline is important for informing later stages of physical therapy, removing unnecessary waste from protocol (eg, steps that do not add value and increase unnecessary utilization without a clear tie to evidence-based medicine and outcomes), and maintaining clear communication with peers, the patient, and their family.

### Theme Number 4: Future Exploration Areas

In order to more accurately diagnose and prevent injury as well as enhance post-injury rehabilitation, new discoveries need to be made in the following areas: (1) investigating cryotherapy to reduce opioid misuse, (2) evaluating electrical stimulation for muscle activation and pain relief, (3) assessing blood flow restriction for improved muscle growth and performance, (4) exploring the use of remote monitoring, especially in challenging environments though step counters, heart rate monitors, etc., and (5) more heavily emphasizing pre-rehabilitation before MSI surgical procedures to better prepare patients for the expectations and important rehabilitative milestones that one should expect. The consensus stated that these areas would allow for additional intramural and extramural grant opportunities, as well as allow care providers to jointly advance care.

## CONCLUSIONS

The Tri-Service meeting was critical for understanding the barriers/challenges to streamlining care in order to jointly improve patient outcomes and satisfaction, improve recovery times and return to duty rates, reduce cost, and decrease unncessary healthcare utilization. The health and well-being of Service Members remain critical factors for our country’s safety and security, as well as readiness in times of military need. The event hosted by MIRROR standardized 11 high-value postoperative protocols and identified key gaps that still need to be closed. All protocols described herein are freely available on (www.mirrorusuhs.org) for private and public sector usage. Further activities are needed to select high-value outcome measures, store these within a common repository, and track data longitudinally to gain new insights to prevent injuries and stop the recurrence of existing ones.
